# Supplementary material for: Trends in Prescription Opioid Use in Motor Vehicle Crash Injuries in the United States: 2014–2018
Source: Int J Environ Res Public Health. 2022 Nov 4;19(21):14445. doi: 10.3390/ijerph192114445 (PMC9657604; doi:10.3390/ijerph192114445)
Supplement: Supplementary file 1 [file ijerph-19-14445-s001.zip › ijerph-1889099-supplementary.pdf]

Supplementary Materials

Table S1. Summary of Emergency Department visits for motor vehicle crashes (MVC) with or without the involvement of prescription opioids.

	Total MVC Injuries (*N* = 142,204)	MVC Injuries Involving Opioids (*N* = 27,275)		MVC Injuries Not Involving Opioids (*N* = 114,929)		*p* values
	**N**	**N or Mean**	**Row % or SD**	**N or Mean**	**Row % or SD**	
Year of injuries						<0.001
2014	20,818	5060	24%	15,758	76%	
2015	33,452	7653	23%	25,799	77%	
2016	36,350	7168	20%	29,182	80%	
2017	27,673	4538	16%	23,135	84%	
2018	23,911	2856	12%	21,055	88%	
Centers for Disease Control and Prevention opioid use guidelines						<0.001
After (>= March 2016)	82,526	13,441	16%	69,085	84%	
Before (< March 2016)	59,678	13,834	23%	45,844	77%	
Age in years	142,204	41	15	38	15	<0.001
Age group (years)						<0.001
18-20	16,557	1876	11%	14,681	89%	
21-24	20,277	2908	14%	17,369	86%	
25-34	29,205	4986	17%	24,219	83%	
35-44	25,817	5630	22%	20,187	78%	
45-54	25,601	6058	24%	19,543	76%	
55-65	20,308	4949	24%	15,359	76%	
> 65	4,439	868	20%	3,571	80%	
Sex						0.829
Male	60,799	11,645	19%	49,154	81%	
Female	81,405	15,630	19%	65,775	81%	
Insurance type						<0.001
Fee for Service	123,541	23,921	19%	99,620	81%	
Encounter	18,663	3354	18%	15,309	82%	
Relation to Employee						<0.001
Employee	81,657	16,362	20%	65,295	80%	
Spouse	25,129	6225	25%	18,904	75%	
Child/Other	35,418	4688	13%	30,730	87%	
Employment Status						<0.001
Active Full Time	106,529	19,682	19%	86,847	82%	
Not Active Full Time	12,244	2452	20%	9,792	80%	
Other/Unknown	23,431	5141	22%	18,290	78%	
Region						<0.001
Northeast Region	14,041	1503	11%	12,538	89%	

North-Central Region	33,837	6002	18%	27,835	82%	
South Region	67,480	14,287	21%	53,193	79%	
West Region	26,846	5483	20%	21,363	80%	
Metropolitan Statistical Area						<0.001
Yes	125,199	23,696	19%	101,503	81%	
No	17,005	3579	21%	13,426	79%	

Table S2. Prevalence and prevalence ratios for prescription opioids in motor vehicle crash (MVC) injuries before vs. after the CDC guidelines, considering transitory periods, US, 2014–2018.

Transitory Period	Before vs. after the CDC Guidelines	Number of MVC Injuries	Percentage of MVC Injuries Involving Opioids	Prevalence Ratio (95% CI)
0	Before	59,678	23%	1 (Reference)
	After	82,526	16%	0.71 (0.70, 0.73)
3 months	Before	59,678	23%	1 (Reference)
	After	73,976	16%	0.69 (0.70, 0.71)
6 months	Before	59,678	23%	1 (Reference)
	After	64,668	15%	0.67 (0.65, 0.68)
12 months	Before	59,678	23%	1 (Reference)
	After	48,002	14%	0.62 (0.60, 0.64)

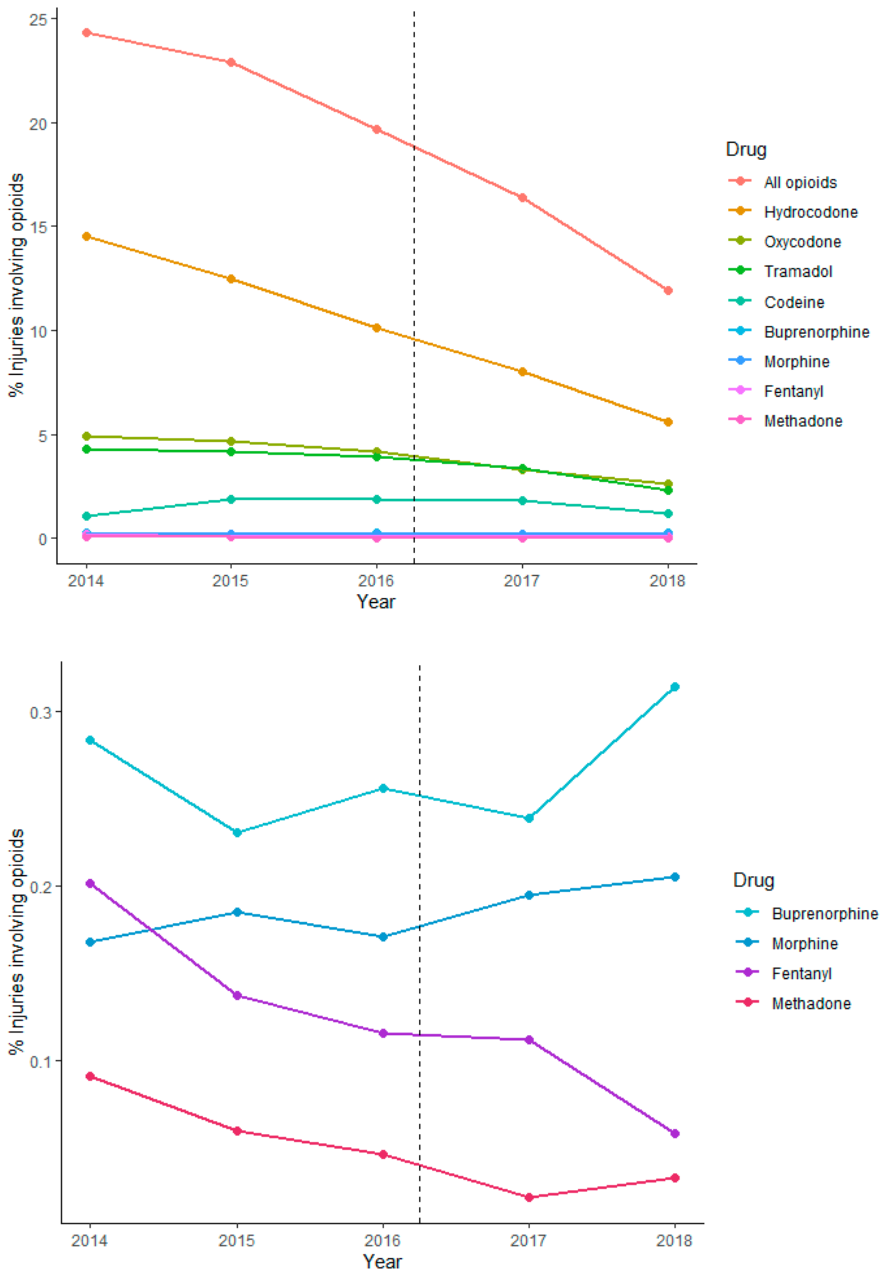

Figure S1. The prevalence of common prescription opioids in motor vehicle crash (MVC) injuries, 2014–2018. Note: the top panel shows all common opioids and the bottom panel shows the opioids with a prevalence < 1%. The vertical dotted line indicates the 2016 release of CDC Guidelines for Prescribing Opioids for Chronic Pain [12].

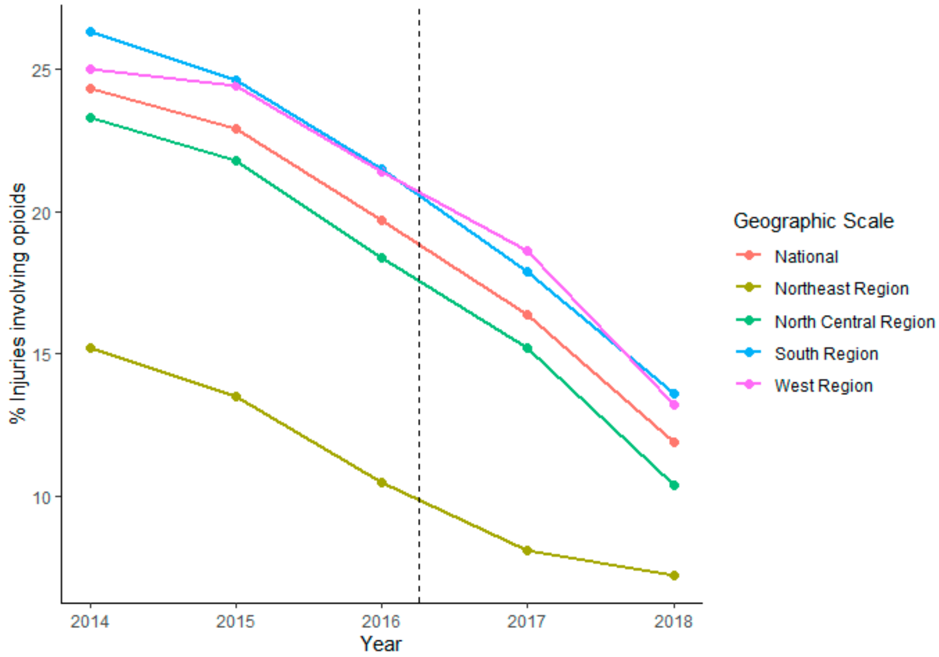

Figure S2. The prevalence of prescription opioids in MVC injuries at national and regional levels, US, 2014–2018. The vertical dotted line indicates the 2016 release of CDC Guidelines for Prescribing Opioids for Chronic Pain [12].
